# Triple‐Phase Interface Engineered Hierarchical Porous Electrode for CO_2_ Electroreduction to Formate

**DOI:** 10.1002/advs.202204472

**Published:** 2022-09-01

**Authors:** Tong Shi, Dong Liu, Ning Liu, Ying Zhang, Hao Feng, Qiang Li

**Affiliations:** ^1^ State Key Laboratory of Multiphase Flow in Power Engineering School of Energy and Power Engineering Xi'an Jiaotong University Xi'an 710049 China; ^2^ MIIT Key Laboratory of Thermal Control of Electronic Equipment School of Energy and Power Engineering Nanjing University of Science and Technology Nanjing 210094 China

**Keywords:** bismuth nanosheets arrays, CO_2_ reduction, formate, hierarchical porous structures, triple‐phases interfaces

## Abstract

The aqueous electrochemical CO_2_ reduction to valuable products is seen as one of the most promising candidates to achieve carbon neutrality yet still suffers from poor selectivity and lower current density. Highly efficient CO_2_ reduction significantly relies on well‐constructed electrode to realize efficient and stable triple‐phase contact of CO_2_, electrolyte, and active sites. Herein, a triple‐phase interface engineering approach featuring the combination of hierarchical porous morphology design and surface modification is presented. A hierarchical porous electrode is constructed by depositing bismuth nanosheet array on copper foam followed by trimethoxy (1H,1H,2H,2H‐heptadecafluorodecyl) silane modification on the nanosheet surface. This electrode not only achieves highly selective and efficient CO_2_ reduction performance with formate selectivity above 90% over wide potentials and a partial current density over −90 mA cm^−2^ in H‐cell but also maintains a superior stability during the long‐term operation. It is demonstrated that this remarkable performance is attributed to the construction of efficient and stable triple‐phase interface. Theoretical calculations also show that the modified surface optimizes the activation path by lowering thermodynamic barriers of the key intermediates *OCHO for the formation of formate during electrochemical CO_2_ reduction.

## Graphical abstract

A triple‐phase interface engineering approach featuring the combination of hierarchical porous morphology design and surface modification is achieved for highly efficient and superiorly stable electrochemical conversion of CO_2_ to formate over wide operating potentials. The average formate selectivity of about 96% is realized in the long‐term operation at 1.2 V vs RHE.

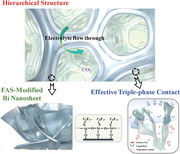

## 1 Introduction

Carbon neutrality is the essential pathway for sustainable development concept of human society. Exploration and utilization of renewable energy to partially replace fossil energy and decrease carbon emission then become urgently for achieving the goal of carbon neutrality and alleviating environmental issues and energy crisis.^[^ ^1^, ^2^ ^]^ Since most of these renewable energies still face temporal and spatial maldistributions as well as intermittence, various strategies in simultaneous conversion and storage of renewable energies into chemical energies toward convenient transportation and utilization then are widely proposed.^[^ ^3^, ^4^, ^5^ ^]^ Among them, electrochemical CO_2_ reduction combined with renewable energy is a promising path to realize carbon neutrality.^[^ ^2^, ^6^, ^7^, ^8^, ^9^, ^10^, ^11^, ^12^, ^13^ ^]^

CO_2_ reduction reaction (CO_2_RR) can produce a variety of valuable products including CO, CH_4_, C_2_H_4_, CH_3_OH, and formate. Among these products, formate is regarded as a promising candidate for industrial application attributing to its high economic benefits and flexible transportation and storage.^[^ ^6^, ^14^ ^]^ Thus, tremendous efforts have been concentrated on the highly selective CO_2_ conversion to formate through the design of electrocatalysts,^[^ ^14^, ^15^ ^]^ where bismuth‐based catalyst is one of the potential choices because of the inherent ability in promoting the intermediates adsorption of CO_2_RR^[^ ^2^, ^8^, ^16^, ^17^, ^18^, ^19^ ^]^ and inhibiting the competitive hydrogen evolution reaction (HER).^[^ ^20^, ^21^ ^]^ However, the CO_2_RR products selectivity, especially at high operating potential, is still a formidable challenge.^[^ ^18^, ^22^, ^23^ ^]^ This is because, first, the solubility of CO_2_ in water is only 0.034 mol L^−1^, making it difficult to maintain a high concentration of CO_2_ near the surface of electrolyte. In addition, the thermodynamic potential of HER is lower than that of CO_2_RR, the pervasive presence of water molecules inevitably makes HER more likely to occur.^[^ ^1^, ^24^, ^25^ ^]^

In principle, CO_2_RR is a triple‐phase heterogeneous reaction, so efficient triple‐phase contact between the gaseous CO_2_ molecules, liquid electrolyte, and solid electrocatalyst active sites is essential to ensure the CO_2_RR activity.^[^ ^16^, ^17^, ^26^, ^27^ ^]^ To date, many studies have sought to modify the electrocatalyst surface with hydrophobic layer to address deficient access to CO_2_ molecules.^[^ ^16^, ^18^, ^26^ ^]^ For example, Wakerley et al.^[^ ^26^ ^]^ developed a bioinspired superhydrophobic Cu dendrite electrode by 1‐octadecanethiol treatment and demonstrated the latent ability in selective CO_2_RR. However, the current density of the modified electrode was significantly decreased due to the concealment of the intrinsic properties of the electrocatalyst, which directly hindered the access of liquid electrolyte to solid active sites.^[^ ^16^, ^17^, ^28^ ^]^ In addition, many have raised the doubt on the stability of these hydrophobic coatings.^[^ ^16^, ^23^, ^28^ ^]^

Herein, we presented a triple‐phase interface engineering approach featuring the combination of hierarchical porous morphology design and surface modification and achieved efficient and selective conversion of CO_2_ to formate. In detail, the bismuth nanosheet array (Bi NSA) was first deposited on a porous Cu foam (Bi NSA@CF), and then an aerophilic network with the functional group of fluoroalkyl chain was decorated on the surface of Bi NSA. The macroscale pores originated from the copper foam provide crisscross microchannels which are in favor of electrolyte transportation. Abundant tiny voids derived by nanosheets array allow more exposure of solid active sites. The aerophilic network enables efficient transfer and adsorption of CO_2_. Thus, the electrode with efficient triple‐phase contact was successfully constructed. Furthermore, theoretical calculation reflects that the introduced functional groups provide better thermodynamic advantage for the generation of the key intermediate *OCHO during the formation of formate. Last but not the least, the aerophilic network also inherently includes Bi—O—Si and Si—O—Si linkages, which stables the triple‐phase interface. By leveraging this approach, selective formate production (selectivity higher than 90%) was achieved over a wide potential window and a partial current density higher than 90 mA cm^−2^ was achieved at the formate selectivity of 98%. This performance is remarkable in H‐type cell. Meanwhile, during the long‐term operation of about 25 h, the electrode maintained a superior stability for both the current density and formate selectivity.

## 2 Results and Discussion

### 2.1 Hierarchical Porous Electrode with Surface Modification

**Scheme 1:**
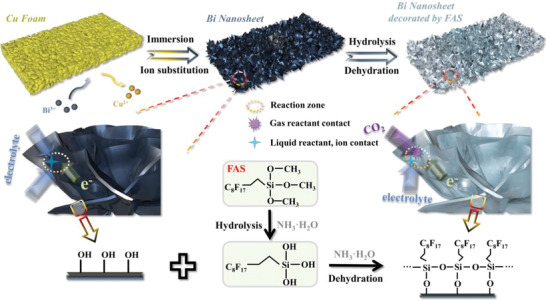
Schematic description of the preparation of hierarchical porous electrode with triple‐phase interface.

The preparation of hierarchical porous electrode Bi NSA @CF with further surface modification, which was denoted as Bi NSA‐TP@CF, was schematically illustrated in **Scheme** **1**. Porous Cu foam was used as the electrode substrate and bismuth nanosheets array was deposited followed by surface modification. For the Bi NSA synthesis, a simple galvanic replacement reaction that motived by the slightly lower potential of Bi^3+^/Bi (0.31 V vs standard hydrogen electrode (SHE)) compared to Cu^2+^/Cu (0.34 V vs SHE) was used as^[^ ^29^ ^]^

(1) $$2Bi^{3+}+3\mathrm{Cu}\to 2\mathrm{Bi}+3Cu^{2+}$$

The surface Cu gradually dissolved and Bi nucleated. These nuclei accumulated and then grew up to construct 2D nanosheets. Subsequently, trimethoxy (1H,1H,2H,2H‐heptadecafluorodecyl) silane (FAS) hydrolysis and dehydration processes under the catalysis of ammonia through solvothermal method were employed to decorate the surface of Bi NSA.^[^ ^30^, ^31^, ^32^ ^]^ As can be seen in Equations (2) and (3), FAS initially underwent the hydrolysis process for the formation of hydroxyl. Then, the consequential dehydration condensation between the hydrolysis products of FAS and the surface hydroxyl of the hydrophilic Bi NSA went on for the formation of aerophilic network.

(2)

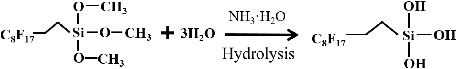



(3)

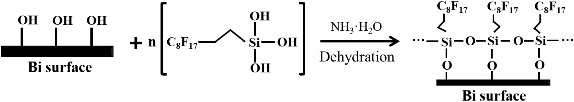




**Scheme 2:**
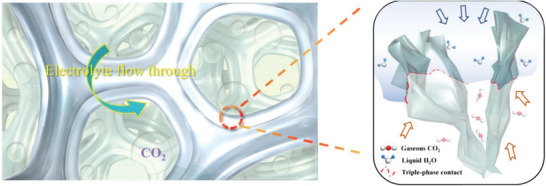
Schematic diagram of triple‐phase contact in Bi NSA‐TP@CF electrode, possessing hierarchical porous structure and FAS surface modification.

Such aerophilic network concurrently allowed the hydrophobic group of long fluoroalkyl chains (—C_8_F_17_) and connecter and stabilizer performed by Bi—O—Si and Si—O—Si linkages. By these, the hierarchical porous electrode with triple‐phase interface was fabricated. As shown in **Scheme** **2**, the constructed hierarchical porous electrode with surface modification inherently allows: 1) macroscale pores and crisscross microchannels for enhancing the gas and liquid reactants transfer; 2) abundant tiny voids in the uniform 2D nanosheets array to provide adequate solid active sites; 3) aerophilic network to guarantee efficient transfer and adsorption of CO_2_. These merits then result in the Bi NSA‐TP@CF electrode allowing an efficient triple‐phase contact between gaseous CO_2_ and liquid electrolyte as well as solid active site, which play positive roles in facilitating the CO_2_RR.

**Figure 1:**
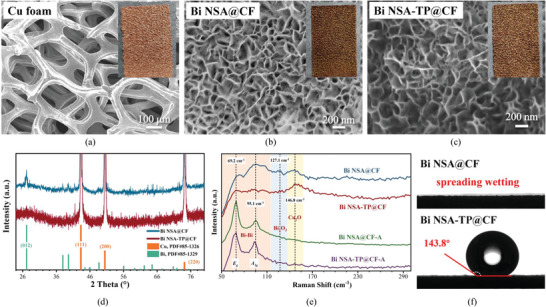
SEM images of a) Cu foam, b) Bi NSA@CF, and c) Bi NSA‐TP@CF electrodes. d) XRD patterns. e) Low wave number Raman spectrums of electrodes before and after operation. f) Static water drop contact angles.

The morphological structures of Cu foam, Bi NSA@CF, and Bi NSA‐TP@CF electrodes are shown in **Figure** **1**a–c. It can be found that the morphology of Bi NSA@CF can be maintained after solvothermal treatment. The porous Cu foam inherently provided plentiful macropores, while the distinct uniform 2D nanosheets array structures also allowed adequate micropores. Such hierarchical pore structure not only emerged abundant active sites for the catalytic reaction but also furnished interconnected microchannels for the reactants transfer.

**Figure 2:**
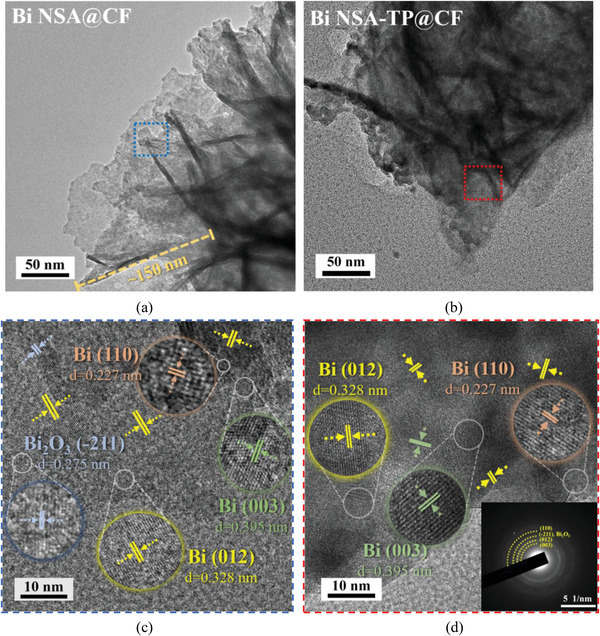
TEM and HRTEM images with marked lattice fringes of a,c) Bi NSA@CF and b,d) Bi NSA‐TP@CF electrodes.

Besides, the precise structure and crystalline feature of Bi NSA@CF and Bi NSA‐TP@CF were also characterized by high‐resolution transmission electron microscopy (HRTEM). The apparent rose‐like morphology of Bi NSA was observed with the vertical length of the individual Bi nanosheet of about 150 nm (**Figure** **2**a). The predictable element distributions of F and Si except Bi and Cu in the energy dispersive spectroscopic (EDS) elemental mapping profile and spectrum results also implied the formation of FAS modified layer on Bi nanosheets (Figures S1 and S2, Supporting Information).^[^ ^29^ ^]^ Therein, obvious Cu signal was also found in the EDS mapping. This result may be ascribed to the following two reasons. First, the Bi nanosheets sample was ultrasonically peeled off from the Cu foam, some Cu components were then peeled off together with the Bi nanosheets. Second, the inadequate cleaning after the galvanic replacement reaction also led to the residual Cu existence on the Bi nanosheets. Various distinct Bi lattice fringes including 0.328 nm (012), 0.395 nm (003), and 0.227 nm (110), were observed in both electrodes. The dominated crystal plane of Bi nanosheets for both samples was Bi (012) (selected area electron diffraction, SAED results in Figure 2d), which were consistent with the X‐ray diffraction (XRD) pattern (Figure 1d). Meanwhile, because of surface oxidation, there also existed a trace of Bi_2_O_3_ and Cu_2_O species in both Bi NSA@CF and Bi NSA‐TP@CF samples (Figure 2c,d). Raman spectroscopy results shown in Figure 1e also marked out the characteristic peaks at 127.1 cm^–1^ (Bi_2_O_3_)^[^ ^33^ ^]^ and 146.8 cm^–1^ (Cu_2_O)^[^ ^34^ ^]^ in both electrodes. However, after a 30 min electroreduction at −1.0 V versus reversible hydrogen electrode (RHE), labeled as Bi NSA@CF‐A and Bi NSA‐TP@CF‐A, *E*_g_ and *A*_1g_ stretching modes of Bi—Bi bond near 69.2 and 95.1 cm^–1^ embodied Bi^0^ phase in both electrodes.^[^ ^20^, ^35^ ^]^ These results indicated that the residual metal oxides on the electrode were soon reduced to the metallic state in the initial stage of the electrochemical reduction process, which further revealed that the main active sites for CO_2_RR were metallic Bi nanosheets rather than the metal oxides.

Furthermore, although the morphological structure and crystalline feature for both Bi NSA@CF and Bi NSA‐TP@CF were similar, the wettability revealed diametrically opposite feature. As shown in Figure 1f, a spreading wetting property was observed for Bi NSA@CF sample, but the contact angle of Bi NSA‐TP@CF sample was over 143.8°. Such excellent hydrophobicity can be attributed to the long alkyl chain and fluorinated group of FAS, which then exposed the potential in capturing gaseous CO_2_ reactant. Meanwhile, to systematically emerge the influence of surface modification on CO_2_ capture and reduction, we have regulated the volume ratio of FAS from 1: 10: 100 to 1: 10: 100 000 (FAS: NH_3_·H_2_O: Ethanol) during electrode preparation process. It can be found that hydrophobic states, even at a definitely low volume ratio, were observed for these Bi NSA‐TP@CF electrodes (Figure S8, Supporting Information), which indicated that the method of FAS modification was reliable to construct excellent hydrophobic surface for CO_2_ capture.

### 2.2 CO_2_RR Performance Evaluation

In this section, CO_2_RR performance of both Bi NSA@CF and Bi NSA‐TP@CF electrodes were evaluated in a commercially available hermetic two‐chamber H‐cell. Prior to the test, electrochemical active surface area (ECSA) presenting by double‐layer capacitances (*C*_dl_) was employed to assess the fair comparison of the two electrodes. Results shown in Figure S3 in the Supporting Information presented well‐fitted values of *C*_dl_ for both Bi NSA@CF (6.22 µF cm^−2^) and Bi NSA‐TP@CF (6.75 µF cm^−2^), indicating that ECSAs of the two electrodes are very close.

**Figure 3:**
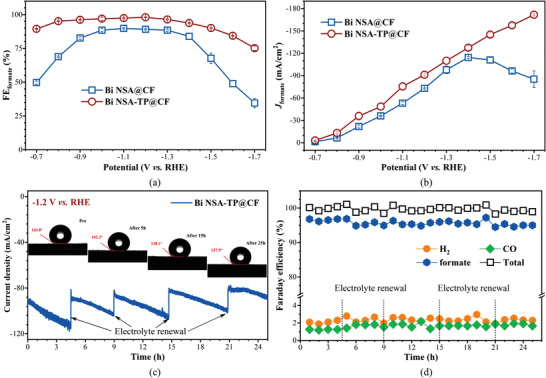
a) Potential‐dependent formate selectivity and b) partial formate current densities of Bi NSA@CF and Bi NSA‐TP@CF electrodes. c) Current density evolution of Bi NSA‐TP@CF electrode at −1.2 V versus RHE (insets are the contact angles at different operating stages) and d) the corresponding Faraday efficiency distributions.

**Figure** **3**a,b summarizes the variations of formate selectivity (FE_formate_) and partial current density (*J*_formate_) of Bi NSA@CF and Bi NSA‐TP@CF electrodes in a wide potential window ranging from −0.7 to −1.7 V versus RHE. Total current density and product Faraday efficiency (FE) at different potentials were shown in Figure S4 in the Supporting Information. Results showed that formate was the only liquid product (Figure S10, Supporting Information) and the formate selectivity of Bi NSA‐TP@CF exhibited significant intensification and the highest FE_formate_ of 98.1% can be obtained at −1.2 V versus RHE (Figure 3a and Figure S4e, Supporting Information), compared with Bi NSA@CF electrode. Especially, FE_formate_ remarkably enhanced from 49.7% to 89.5% at low overpotential (−0.7 V vs RHE) and from 34.6% to 75.1% at high overpotential (−1.7 V vs RHE). Moreover, *J*_formate_ of Bi NSA‐TP@CF electrode was gradually intensified with increasing operating potential and it can still approach an outstanding *J*_formate_ over −170 mA cm^−2^ at relatively high potential of −1.7 V versus RHE (Figure 3b). While for Bi NSA@CF, *J*_formate_ was initially intensified, then significantly decreased from −114.5 mA cm^−2^ (−1.4 V vs RHE) to −85.3 mA cm^−2^ (−1.7 V vs RHE) ascribing to the competitive hydrogen evolution side reaction (Figure 3b and Figure S4f, Supporting Information). Besides, the CO_2_RR activities of both Bi NSA @CF and Bi NSA‐TP@CF electrodes were also performed by linear sweep voltammetry (LSV) tests in a CO_2_ saturated 0.5 m KHCO_3_ electrolyte. As shown in Figure S5 in the Supporting Information, the current density of Bi NSA‐TP@CF electrode revealed slightly higher than that of Bi NSA@CF in the voltage window from 0 V versus RHE to −1.39 V versus RHE. This disparity may be ascribed to the slightly increased ECSA of Bi NSA‐TP@CF electrode (Figure S3, Supporting Information), which then exposed more active sites for the cathodic reduction reaction. However, at more negative potentials than −1.39 V versus RHE, the increasingly severe side reaction of hydrogen evolution (Figure 3a,b and Figure S4f, Supporting Information) resulted in the Bi NSA@CF electrode showing a higher current density than that of Bi NSA‐TP@CF electrode. The formate partial current density variation trend with the operating potential was employed for the onset potential identification of CO_2_ to formate process. As shown in Figure S6 in the Supporting Information, using 10 mA cm^−2^ as the standard of onset potential, there are obvious gap between Bi NSA@CF (−0.823 V) and Bi NSA‐TP@CF (−0.769 V), which indicated that the proposed Bi NSA‐TP@CF electrode presented thermodynamic advantage in electrochemical CO_2_ reduction to formate. For comprehensive comparison, the CO_2_RR performance of commercial Bi powder catalyst has also been examined and the results were shown in Figure S7 in the Supporting Information. For both current density and formate selectivity, the proposed Bi NSA‐TP@CF electrode can present remarkable intensification compared with commercial Bi powder electrode. This performance improvement is particularly evident at high operating potentials, where at −1.7 V versus RHE, the FE_formate_ and *J*_formate_ of Bi powder electrode were only 21.15% and −64.34 mA cm^−1^,^[^ ^2^ ^]^ respectively, while they significantly increased to 75.13% and −224.30 mA cm^−2^ for Bi NSA‐TP@CF electrode.

Moreover, the influence of FAS volume ratio on the CO_2_RR performance was also investigated at the operating potential of −1.2 V versus RHE and the results were shown in Figure S9 in the Supporting Information. As shown, although the wettability (≈144.5°, Figure S8, Supporting Information) and total current density (≈94.2 mA cm^−1^,^[^ ^2^ ^]^ Figure S9, Supporting Information) of each Bi NSA‐TP@CF sample were nearly the same, due to the incomplete surface modification or less functional groups on the electrode, both FE_formate_ and *J*_formate_ slightly deceased with the decrease in the volume ratio of FAS. Even though, each Bi NSA‐TP@CF electrode still presented better CO_2_RR performance compared with Bi NSA@CF electrode. These results further indicated that the construction of triple‐phase interface on the electrode surface can play a vital role in facilitating the current density and selectivity of CO_2_ reduction. Furthermore, some other metal substrates were also employed for the growth of Bi nanocatalysts, it can be seen that scattered Bi nanosheets or nanodendrites can also grow on these substrates through galvanic replacement reaction method (Figure S12, Supporting Information) and well formate selectivity of about 82.1% at the operating potential of −0.9 V versus RHE was also obtained in the electrode of Bi grown on Zn foil (Figure S13, Supporting Information), while it is well known that the Zn catalyst possesses poor activity for CO_2_‐to‐formate electrochemical reduction.^[^ ^36^, ^37^ ^]^

#### 2.2.1 Stability during Long‐Term Operation

To evaluate the stability of the proposed Bi NSA‐TP@CF electrode, long‐term CO_2_RR over 25 h was carried out through controlled potential electrolysis at the potential of −1.2 V versus RHE. Results in Figure 3c show that the electrode achieved an average current density of about −94 mA cm^−2^ during the long‐term operation and maintained the hydrophobic state with an almost constant contact angel of about 140° (insets in Figure 3c). Such remarkable performances are mainly ascribed to the kinetic favor brought by hierarchical porous morphology and triple‐phase interface construction; the sufficient reactants (CO_2_ molecules and protons) contact in active sites then makes the catalyst activity fully displayed in higher operating potential. While the current density was slightly increased with the operating time during each period of electrolyte renewal. This increase may attribute to the continuous consumption of protons, which then gradually increased the pH value of the electrolyte and thus led to more negative potential at the electrode.^[^ ^16^ ^]^ Once the electrolyte was renewed, this increase vanished. Besides, the product selectivity during the long‐term operation was summarized and the results were shown in Figure 3d. As shown, both the FE_formate_ and FE_CO_ as well as FE_H2_ remained basically unchanged, and the average FE_formate_ of about 96% was realized. The scanning electron microscope (SEM) characterizations of Bi NSA‐TP@CF after stability test have been carried out in Figure S11 in the Supporting Information, it can be seen that although the 2D nanosheet array structure was annihilated to form nanoparticles, the hierarchical porous structure and surface aerophilicity can still be maintained (Figure 3c), so the superior stability could be realized.

#### 2.2.2 State‐of‐the‐Art Performance Comparison

**Figure 4:**
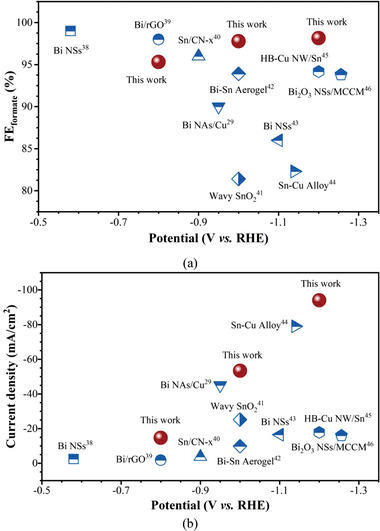
State‐of‐the‐art CO_2_RR performance comparison for a) formate selectivity and b) current density.

Furthermore, we have also made a brief summary of recent reports on electrochemical CO_2_ conversion to formate and compared the CO_2_RR performance between the electrodes reported in literature and in this work (Table S1, Supporting Information). **Figure** **4**a,b compares the formate selectivity and current density of some works in Table S1 in the Supporting Information, respectively. It can be found that, compared with previous works,^[^ ^29^, ^38^, ^39^, ^40^, ^41^, ^42^, ^43^, ^44^, ^45^, ^46^ ^]^ our proposed Bi NSA‐TP@CF electrode not only emerged the superior electrochemical CO_2_RR performance on selectivity and partial current density of formate but also showed excellent stability during long‐term CO_2_ conversion (Table S1, Supporting Information). These results further demonstrated the conspicuous potential of the proposed Bi NSA‐TP@CF electrode for highly efficient and stable electrochemical CO_2_ conversion to valuable products.

### 2.3 Underlying Mechanism Analysis

**Figure 5:**
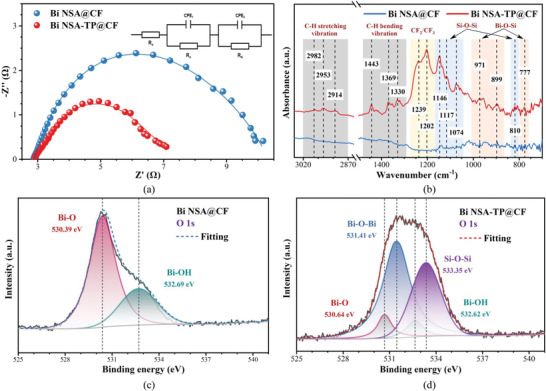
a) Nyquist plots and b) FT‐IR spectrums of Bi NSA@CF and Bi NSA‐TP@CF electrodes. XPS O 1s spectral region recorded and fitting curves for c) Bi NSA@CF and d) Bi NSA‐TP@CF electrodes.

To clarify the origin of enhanced CO_2_RR performance for our proposed electrode, the underlying mechanism was discussed in this section. Since highly efficient and selective CO_2_RR significantly relies on efficient triple‐phase contact of CO_2_, electrolyte and active sites,^[^ ^17^ ^]^ the transfer resistance and surface property was firstly discussed. **Figure** **5**a shows the Nyquist plots and corresponding curves fitted by equivalent circuit (inset in Figure 5a), where *R*_Ω_, *R*_c_, *R*_d_, and CPE represent the ohmic resistance, charge transfer resistance, diffusion transfer resistance and constant phase element, respectively. Fitting parameters of the equivalent circuit shown in Table S2 in the Supporting Information revealed that both the charge transfer resistance (2.58 Ω) and diffusion transfer resistance (1.95 Ω) of Bi NSA‐TP@CF were smaller than that of Bi NSA@CF electrode (*R*_c_ = 3.41 Ω, *R*_d_ = 4.05 Ω). This decrease in transfer resistances could be ascribed to the synergetic effect between the hierarchical porous structure and surface aerophilicity of the proposed electrode, which not only lowered the overpotential during CO_2_RR but also guaranteed efficient ion and CO_2_ supply to construct more triple‐phase contact.^[^ ^16^, ^45^ ^]^ Thus, both higher CO_2_RR product selectivity and partial current density were achieved, especially at higher operating potential (Figure 3a,b and Figure S4, Supporting Information).

Figure 5b shows the Fourier transform infrared (FT‐IR) spectrum of the two electrodes. Compared with Bi NSA@CF, some significant characteristic absorption peaks at 777, 899, and 971 cm^–1^ assigned to the Bi—O—Si bond connecting Bi nanosheets and FAS^[^ ^47^, ^48^ ^]^ and 810, 1074, 1117, and 1146 cm^–1^ attributed to symmetric, asymmetric and various stretching modes of Si—O—Si links^[^ ^30^, ^31^, ^49^, ^50^, ^51^, ^52^ ^]^ were identified for Bi NSA‐TP@CF electrode. These crisscross connections not only stabilized aerophilic network but also allowed plentiful synergetic sites for protons adsorption.^[^ ^53^ ^]^ Besides, strong vibrational peaks centered at 1202 and 1239 cm^–1^ were distinguished as C—F bond signal in CF_2_/CF_3_ originated from FAS property.^[^ ^49^, ^51^ ^]^ This intrinsic hydrophobic group guaranteed the aerophilicity of the Bi NSA‐TP@CF electrode, which can promote the CO_2_ capture. The same results can also be verified by the X‐ray photoelectron spectroscopy (XPS) spectra of the two samples (see Figure 5c,d and Figure S14, Supporting Information). As shown, besides the Bi—O peak and Bi—OH peak in Bi NSA (Figure 5c),^[^ ^54^, ^55^, ^56^ ^]^ two additional characteristic peaks at 531.41 and 533.45 eV corresponding to Bi—O—Si bond and Si—O—Si bond^[^ ^30^, ^48^, ^57^ ^]^ were also found from the O 1s spectral of Bi NSA‐TP@CF (Figure 5d). Quantitative results in Table S3 in the Supporting Information also imply that the proportion of both Bi—O and Bi—OH significantly decreases while Bi—O—Si and Si—O—Si forms. These chemical bondings then can not only play a crucial role in connecting and stabilizing the aerophilic network on the Bi nanosheets but also perform as possible adsorption sites for extra protons to optimize reaction kinetics during CO_2_ electroreduction reaction.^[^ ^53^, ^58^, ^59^, ^60^ ^]^ As a result, a high and stable FE_formate_ and current density during the long‐term operation can be obtained (Figure 3c,d).

**Figure 6:**
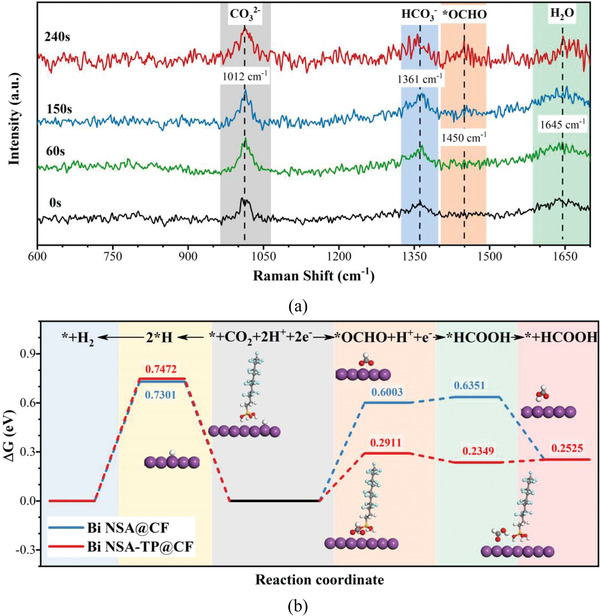
a) Typical in situ Raman spectra of CO_2_RR at −0.9 V versus RHE. b) Gibbs free energies of reaction pathways and corresponding intermediates over Bi NSA@CF and Bi NSA‐TP@CF electrodes.

Furthermore, in situ Raman spectroscopy and density functional theory (DFT) calculation of both Bi NSA@CF and Bi NSA‐TP@CF electrodes were also utilized to deepen the understanding of the enhancement of CO_2_RR performance. In general, for CO_2_‐to‐formate reduction, *OCHO intermediate is the energetically favorable choice for formate formation.^[^ ^61^, ^62^ ^]^ Our in situ Raman results also demonstrated this (**Figure** **6**a). The intrinsic peaks at about 1012, 1361, and 1645 cm^–1^ contributing to CO_3_^2^,^[^ ^63^, ^64^ ^] –^HCO_3_^–^,^[^ ^65^, ^66^ ^]^ and H_2_O^[^ ^63^, ^67^ ^]^ molecule were initially identified. Then, the emerging peak at about 1450 cm^–1^ for *OCHO was also recognized in the CO_2_‐to‐formate reduction process.^[^ ^67^, ^68^ ^]^

The calculated Gibbs free energies during CO_2_RR and side HER processes for the two electrodes show in Figure 6b. As shown, first, for both Bi NSA@CF and Bi NSA‐TP@CF, the formation of *OCHO is the rate‐determine‐step (RDS), while the critical thermodynamic barrier significant decrease from 0.6003 eV of Bi interface to 0.2911 eV of decorated Bi interface. Then, for the second hydrogenation step to *HCOOH, the modified Bi interface presents a downhill reaction whereas a small uphill energy of about 0.035 eV is necessary at the pristine Bi interface. Moreover, compared with the Gibbs free energy of RDSs in CO_2_RR (i.e., the formation of *OCHO) and side HER (i.e., the formation of *H), the disparity for Bi interface is about 0.13 eV while it remarkably increased to about 0.46 eV for Bi NSA TP/CF. These results further reveal the better intrinsic reaction kinetics of the proposed Bi NSA‐TP@CF electrode, which presented the better formate selectivity and partial current density at lower overpotentials (Figure 3 and Figure S4, Supporting Information).

## 3 Conclusion

In summary, we have presented an FAS‐modified hierarchical porous Bi‐based electrode for highly selective and efficient electroreduction of CO_2_ to formate. Ascribing to the hierarchical porous structure with Bi nanosheet array on Cu foam, more active sites and lower transfer resistance were guaranteed. The aerophilic network from FAS modification inherently includes functional groups of hydrophobic fluoroalkyl chains to promote CO_2_ adsorption and transfer as well as Bi—O—Si and Si—O—Si linkages to stabilize the modification layer. Thus, an efficient and stable triple‐phase interface was realized. Furthermore, we also revealed that the FAS modification lowered the thermodynamic barrier of the key intermediate *OCHO during CO_2_‐to‐formate reduction. As a result, state‐of‐the‐art selective, efficient, and stable performance of CO_2_ reduction to formate has been achieved. The combination of hierarchical porous structure design and surface modification opens up an appealing approach for triple‐phase interface engineering.

## 4 Experimental Section

### Electrode Fabrication

The fabrication of the hierarchical porous electrode with triple‐phase interface includes two steps. The first step is the synthesis of vertically aligned Bi nanosheets array (Bi NSA) on porous Cu foam. Herein, previously reported galvanic replacement reaction method was utilized for the Bi NSA preparation.^[^ ^29^ ^]^ Initially, 0.253 g (0.02 m) bismuth chloride (BiCl_3_) was dissolved in 40 mL dimethyl sulfoxide with 30 min stirring. The precleaned Cu foam with the width of 10 mm and length of 20 mm was then soaked in above solution for galvanic replacement reaction between metallic Cu and Bi^3+^ ions at room temperature for 24 h. The obtained electrodes were then rinsed with deionized water (18.2 MΩ cm^−1^) to remove the residual solution and dried with nitrogen flow. By these, Bi NSA was synthesized on porous Cu foam, and this electrode was denoted as Bi NSA@CF.

The second step is the construction of triple‐phase interface on the surface of Bi NSA@CF obtained above. The solvothermal method was conducted in this process by immersing the obtained electrode in the pre‐prepared bath solution for 1 h at the temperature of 70 °C, where the bath solution consisted of trimethoxy (1H,1H,2H,2H‐heptadecafluorodecyl) silane (FAS), ammonia (25 wt%) and ethanol with the volume ratio of 1: 10: 100. After that, the hierarchical porous electrode with stable triple‐phase interface was prepared, which was labeled as Bi NSA‐TP@CF.

### Material Characterization

Electrode morphologies were characterized using a field‐emission scanning electron microscopy (Zeiss Gemini 300, Germany). X‐ray diffraction method (Bruker D8 Advance, Germany) was used to investigate the phase composition of the electrocatalysts at a scan speed of 2° min^−1^ from 20° to 80°. HRTEM and SAED were performed on JEOL JEM‐F200 at an acceleration voltage of 200 kV. Corresponding element mapping and energy spectrum were acquired by JED‐2300T. Mo grid was selected for avoiding the possible Cu contamination from normal Cu grid. FT‐IR spectroscopy was equipped with a diffuse‐reflectance cell and collected by Thermo Scientific Nicolet iS50 at room temperature. Surface composition and chemical states of the prepared electrodes were measured by XPS, which were acquired by a Thermo Scientific ESCALAB 250Xi spectrometer equipped with a 150 W monochromatic Al‐K radiation. Contact angles of drops on electrodes were measured by the sessile drop method (RAMÉ‐HART 290‐U1, USA). Raman spectra were obtained using Horiba LabRam HR Evolution Raman spectrometer. The wavelength and power of the laser were set at 532 nm and 2.5 mW, respectively.

### Electrochemical Measurements

All the electrochemical performances of working electrodes were evaluated in a commercially available hermetic two‐chamber H‐type cell. The anodic chamber and cathodic chamber were separated by a Nafion 117 membrane. CO_2_‐saturated 0.5 m KHCO_3_ aqueous solution was used as electrolyte for both anodic and cathodic chambers. The prepared electrodes that controlled with the geometric area of 10 mm × 10 mm were used as the cathode, an Ag/AgCl electrode (soak in saturated KCl) and a platinum sheet electrode were chosen as reference electrode and anode, respectively. In all CO_2_RR tests, high purity CO_2_ gas was continuously bubbled into the cathode chamber with the flow rate of 20 sccm using a gas mass flow controller (FMA‐2606A‐I, Omega, USA). A stirring magneton was placed at the bottom of the cathode chamber.

All electrochemical measurements were equipped with a standard three‐electrode system and carried out by a CHI 660E (CH Instruments, Shanghai) electrochemical workstation except that the electrochemical impedance spectroscopy (EIS) was performed on a Zennium Pro electrochemical workstation (Zahner, Germany). LSV curves of different electrodes were obtained in a CO_2_‐saturated 0.5 m KHCO_3_ electrolyte. The voltage window ranged from 0.35 to −1.8 V versus RHE, and the scan rate was set at 0.1 V s^−1^. Cyclic voltammetry (CV) test was operated to reveal the ECSA in CO_2_‐saturated 0.5 m KHCO_3_ electrolyte. The sweep range in the non‐Faraday process potential interval was selected from −0.27 to −0.37 V versus Ag/AgCl with the scans rates ranging from 20 to 120 mV s^−1^. The slopes of linear fittings were generated by the plots of capacitive current densities (Δ*j* = *j*_a_ − *j*_c_, where *j*_a_ and *j*_c_ are the anodic and cathodic current densities, respectively) that calculated from CV curves at −0.32 V (vs Ag/AgCl) against scan rates. Such slopes represented the electrochemical double‐layer capacitances (C_dl_), which is proportion to the values of ECSA. The EIS measurements were carried out at operating voltage of −1.2 V versus RHE with a frequency ranged from 100 kHz to 1 Hz and an amplitude of 5 mV. All recorded potentials were standardized to RHE according to the Nernst equation

(4) Potential(VvsRHE)=Potential(vsAg/AgCl)+0.197+0.059pH

where the pH value in above formula equal to 7.4 sustainably in CO_2_‐saturated KHCO_3_ catholyte with continuous CO_2_ gas bubbling.

### Products Analysis

The gaseous CO_2_RR products were collected by a gas‐collecting bag. The bag was connected to the outlet of the cathodic chamber. Gaseous products were injected into an Agilent 7890B gas chromatograph to analyze the component distribution. The liquid products were taken out from the cathodic chamber. The obtained liquid was first diluted with 0.5 m sulfuric acid for the neutralization of bicarbonate. Then, the liquid CO_2_RR products in the neutralized liquid was quantified by an Agilent 1260 infinity II liquid chromatograph equipped with an Agilent Hi‐plex H column and an Agilent variable wavelength detector. Meanwhile, to further identify the liquid products, the liquid NMR spectrum was also measured by Bruker AVANCE III 500 MHz.

The FE was calculated by the following equation

(5) $$\mathrm{FE}=\frac{\mathrm{znF}}{Q}\times 100\%$$

where *z* is the transferred electrons to per mole product, *n* (mol) is the mole of this product, *F* = 96 485 C mol^−1^ is the Faraday constant, *Q* (C) is the total charge, which was derived from the time integral of current.

### Theoretical Calculations

The Gibbs free energies of the reactants, intermediates, and products during CO_2_ reduction were calculated using DFT via Vienna ab initio simulation program (VASP).^[^ ^69^, ^70^ ^]^ The Perdew–Burke–Ernzerhof^[^ ^71^ ^]^ functional within generalized gradient approximation^[^ ^72^ ^]^ was utilized in this study. Bi(012) was chosen because it is the main facet over the catalyst according to characterization results of XRD, HRTEM, and SAED tests. A plane‐wave‐basis cutoff energy of 400 eV was used in geometry optimization. The convergence criterion of energy was set to 10^–6^ eV in this study and the structure is considered to be converged once the force is lower than 0.02 eV Å^−1^. The free energies were calculated within p(2 × 2) supercells of the single‐crystal Bi(012) surface, with the upper two atomic layers relaxed. And a Monkhorst–Pack *k*‐point grid of 2 × 2 × 1 was employed in this periodic structure. The vacuum space between slabs was set to 15 Å to minimize the interactions along the *z* direction. The correction of Gibbs free energy includes zero‐point energies (ZPE), entropies, and heat capacities. Therefore, the change in free energy for each elementary step is given by

(6) $$\Delta G=\Delta E+\Delta ZPE-T\Delta S+\Delta \int C_{V}dT$$

where Δ*E* is the DFT energy for the system, Δ∫*C*_V_ *dT* is the heat capacity, and the Δ*S* is entropy. The computational hydrogen electrode method was employed such that the free energy of 1/2 H_2_ is equal to that of (H^+^ + e^–^) at 0 V versus RHE, and the energy of an electron will be shifted by −eU.^[^ ^73^ ^]^

## Conflict of Interest

The authors declare no conflict of interest.

## Author Contributions

T.S. and D.L. contributed equally to this work. The author contribution is as follows: conceptualization, investigation, writing original draft (T.S.); investigation, data analysis, writing original draft (D.L.); theoretical calculation, writing original draft (N.L.); investigation, writing—original draft (Y.Z.); conceptualization, methodology, investigation, resources, review and editing (H.F.); and supervision, resources, review and editing (Q.L.).

## Supporting information

Supporting Information

## Data Availability

The data that support the findings of this study are available from the corresponding author upon reasonable request.
