# Supplementary material for: Triple‐Phase Interface Engineered Hierarchical Porous Electrode for CO2 Electroreduction to Formate
Source: Adv Sci (Weinh). 2022 Sep 1;9(30):2204472. doi: 10.1002/advs.202204472 (PMC9596843; doi:10.1002/advs.202204472)
Supplement: Supplementary file 1 — Supporting Information [file ADVS-9-2204472-s001.pdf]

ADVANCED SCIENCE
Open Access

Supporting Information

for *Adv. Sci.*, DOI 10.1002/advs.202204472

Triple-Phase Interface Engineered Hierarchical Porous Electrode for CO_2 Electroreduction to Formate

Tong Shi, Dong Liu, Ning Liu, Ying Zhang, Hao Feng and Qiang Li**

Supporting Information for

Triple-Phase Interface Engineered Hierarchical Porous Electrode for CO_2 Electroreduction to Formate

Tong Shi [a, b,1], Dong Liu [a,1], Ning Liu [a], Ying Zhang [a], Hao Feng [a,*], Qiang Li [a,b,*]

[a] State Key Laboratory of Multiphase Flow in Power Engineering, School of Energy and Power Engineering, Xi'an Jiaotong University, Xi'an 710049, China

[b] MIIT Key Laboratory of Thermal Control of Electronic Equipment, School of Energy and Power Engineering, Nanjing University of Science and Technology, Nanjing 210094, China

[1] These authors contributed equally

*Corresponding Author:

fenghao@njust.edu.cn (Hao Feng); liqiang@njust.edu.cn (Qiang Li)

Contents

1. Supplementary Figures S1 to S14

2. Supplementary Tables S1 and S3

SUPPLENMENTARY FIGURES

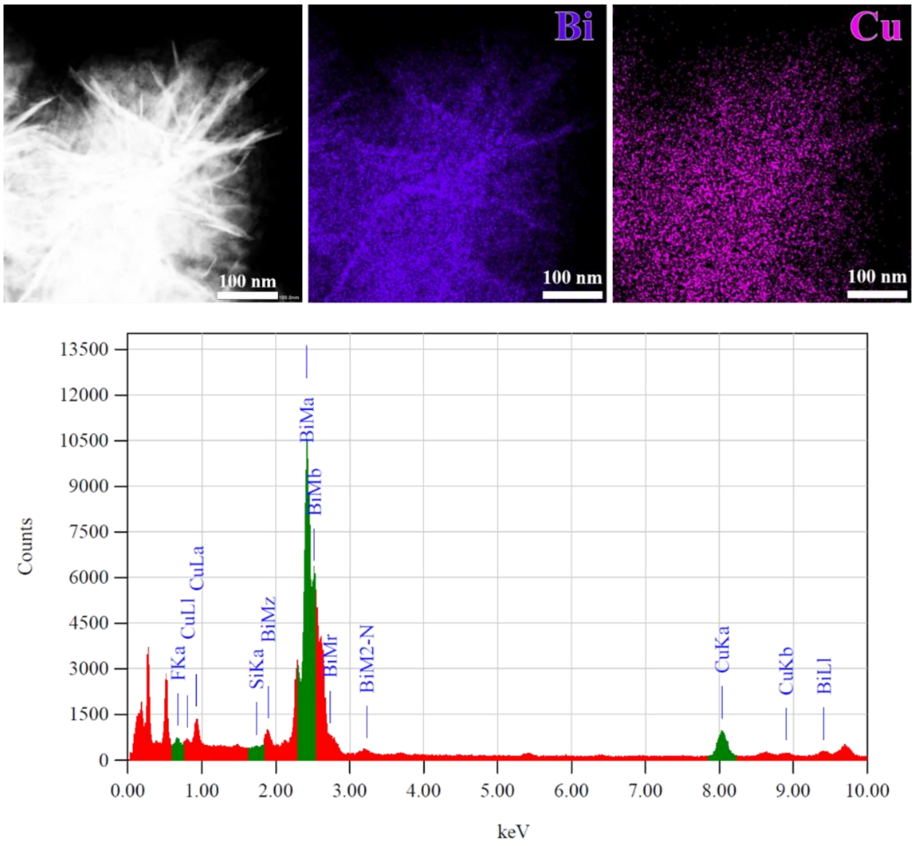

Figure S1 TEM image with corresponding element distribution and EDS spectrum of Bi NSA@CF electrode.

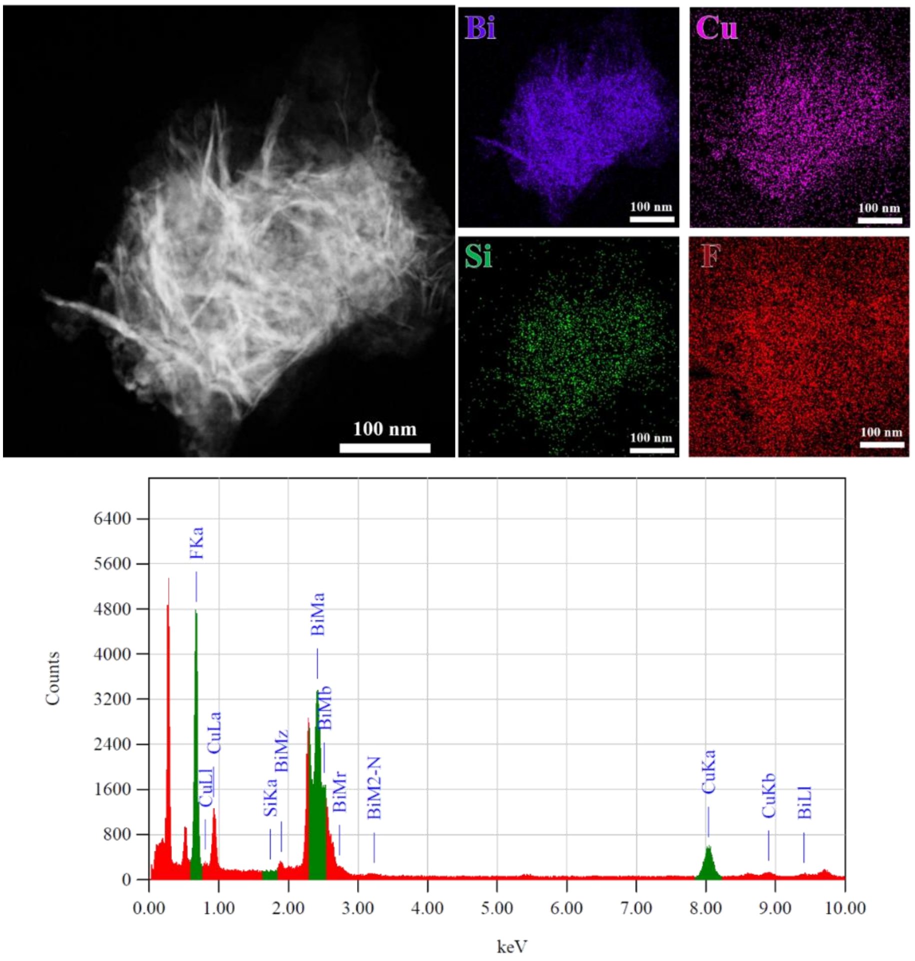

Figure S2 TEM image with corresponding element distribution and EDS spectrum of Bi NSA-TP@CF electrode.

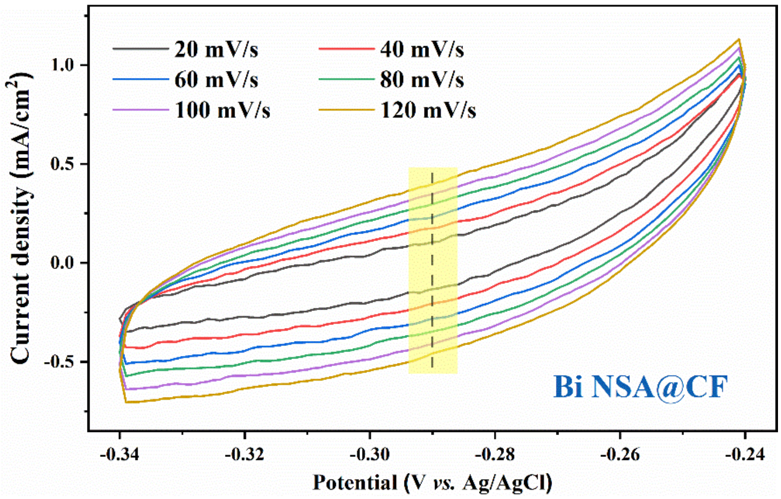

(a)

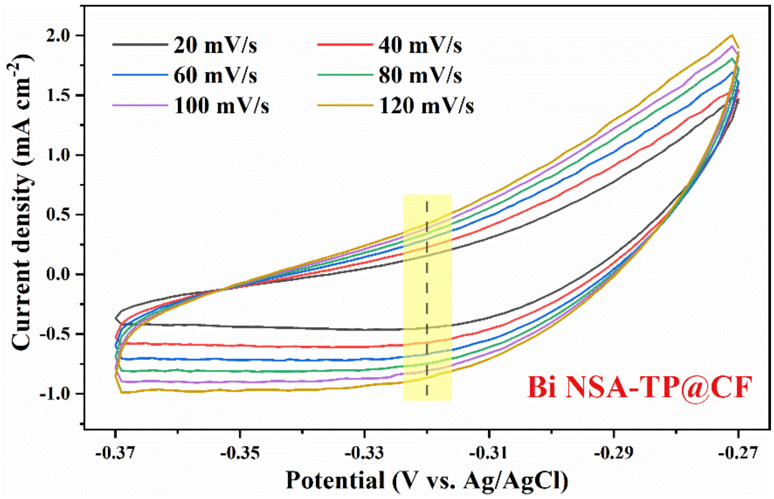

(b)

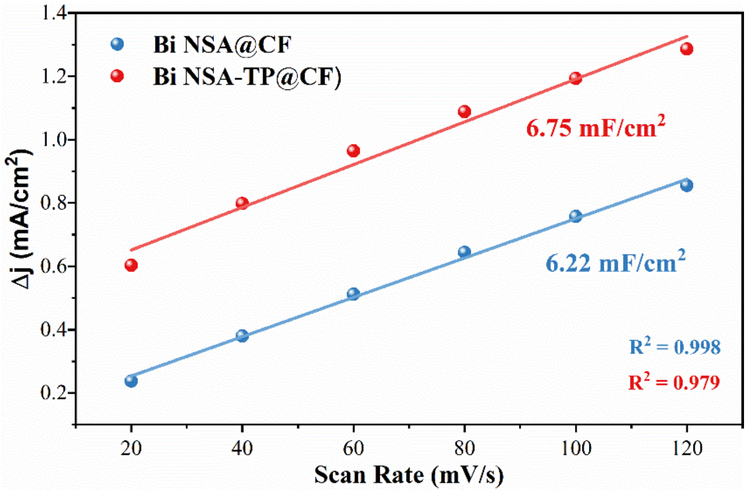

(c)

Figure S3 CV curves of (a) Bi NSA@CF and (b) Bi NSA-TP@CF electrodes with the scan rates from 20 to 120 mV/s and (c) the ECSA comparison.

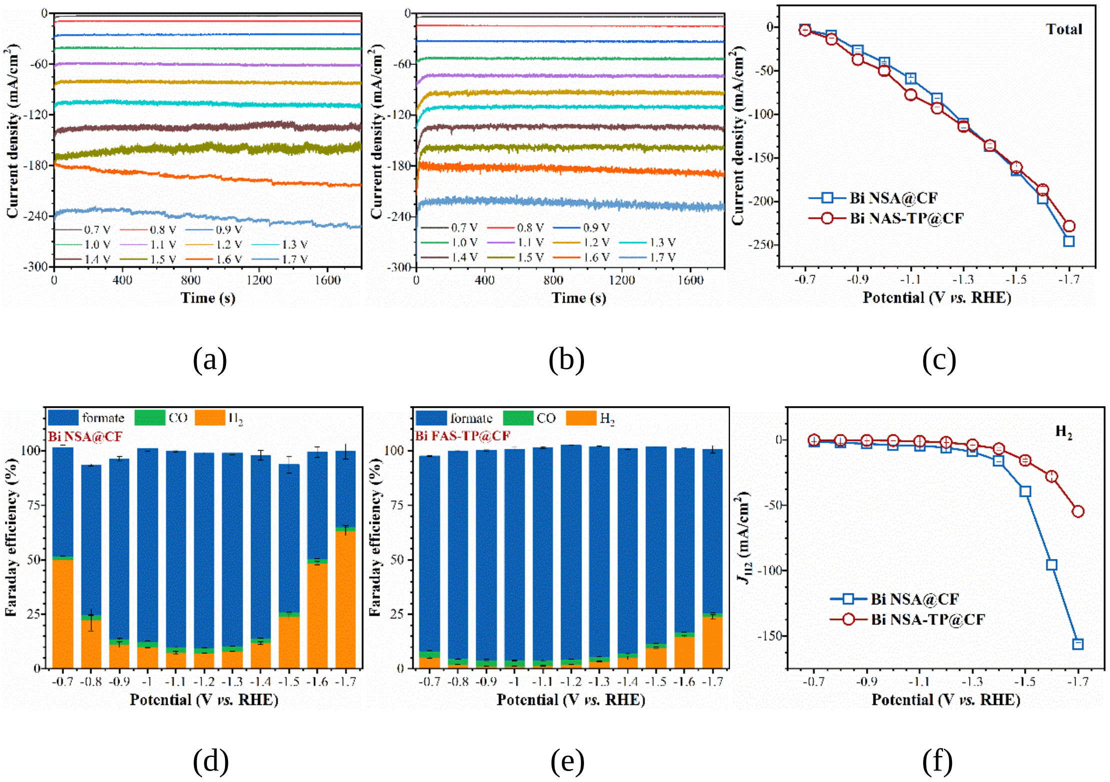

Figure S4 (a) Current density evolution of (a) Bi NSA@CF and (b) Bi NSA-TP@CF electrodes at different operating potential. (c) Current density comparison. Products selectivity of (d) Bi NSA@CF and (e) Bi NSA-TP@CF electrodes. (f) Partial H_2 current density.

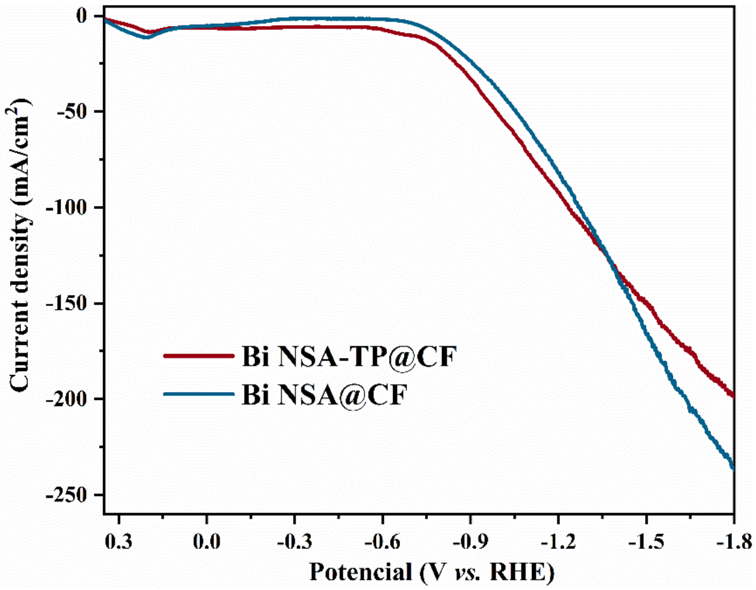

Figure S5 Comparison of Bi NSA@CF and Bi NSA-TP@CF electrodes in LSV performance.

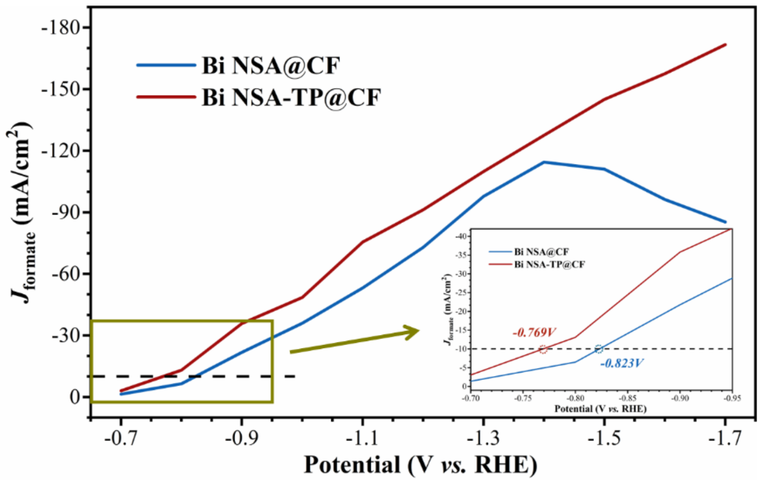

Figure S6 Variations of formate partial current densities for Bi NSA@CF and Bi NSA-TP@CF electrodes with the increase of operating potential.

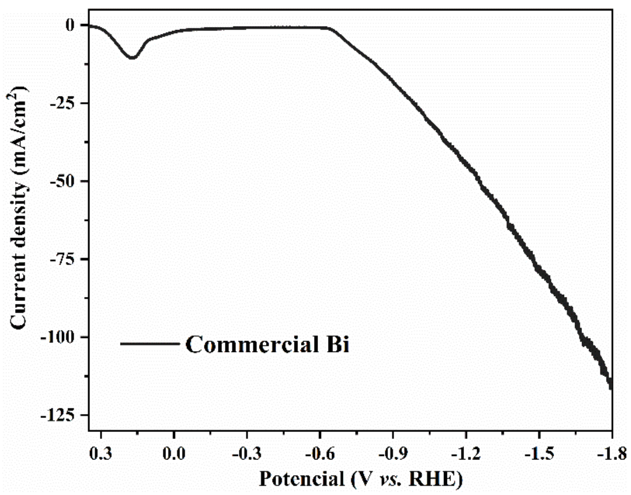

(a)

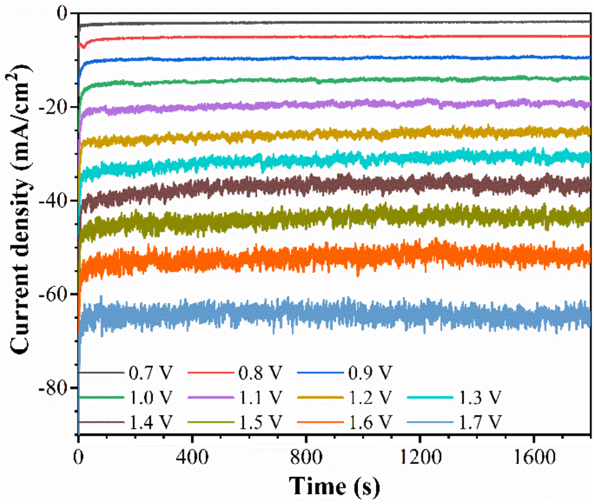

(b)

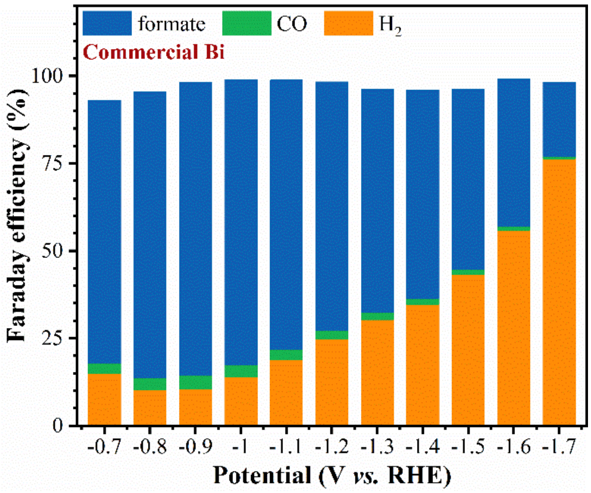

(c)

Figure S7 (a) LSV performance, (b) current densities at different operating potential and (c) corresponding product selectivity of commercial Bi powder.

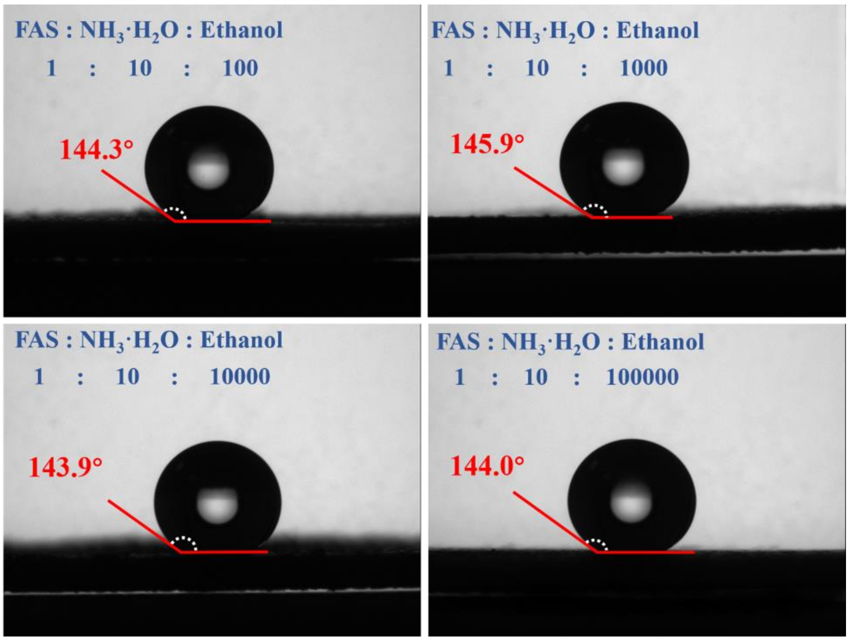

Figure S8 Contact angles of the electrodes prepared with different volume ratios ($FAS: NH_3{\cdot}H_2O$: Ethanol)

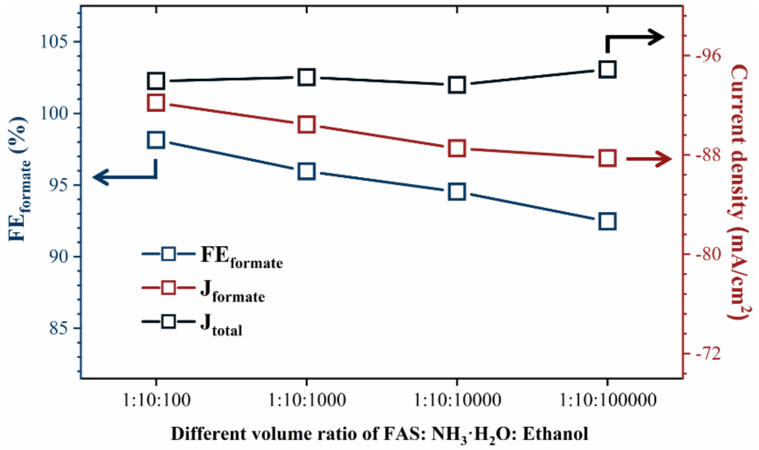

Figure S9 The formate FE (blue) and current density ($J_{formate}$, red; J_{total}, black) variations of Bi NSA-TP@CF electrodes with different volume ratios at -1.2V vs. RHE.

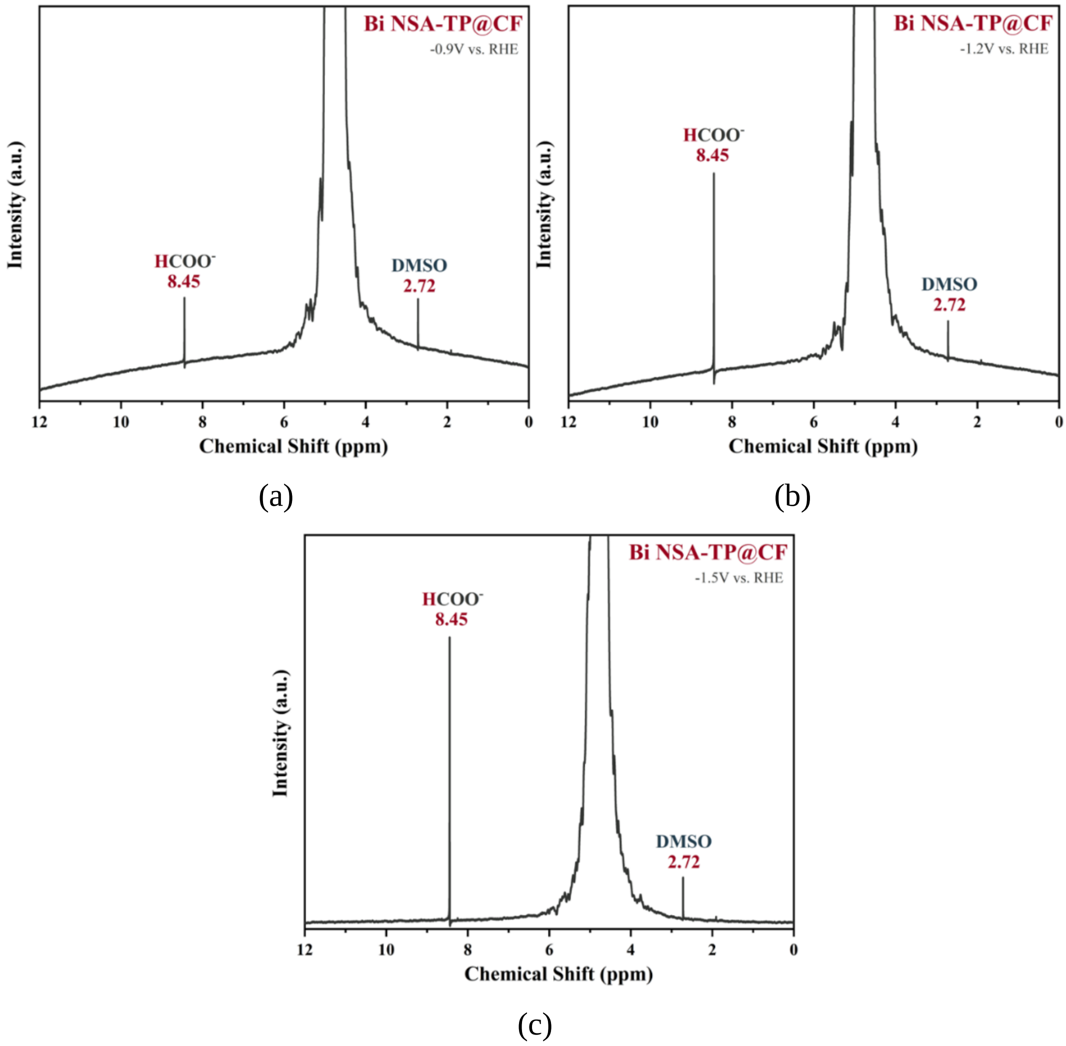

Figure S10 Liquid product NMR spectra of Bi NSA-TP@CF electrode at different operating potentials of (a) -0.9V, (b) -1.2V and (c) -1.5V vs. RHE after 30min reaction in CO2-saturated 0.5 M $KHCO_3$.

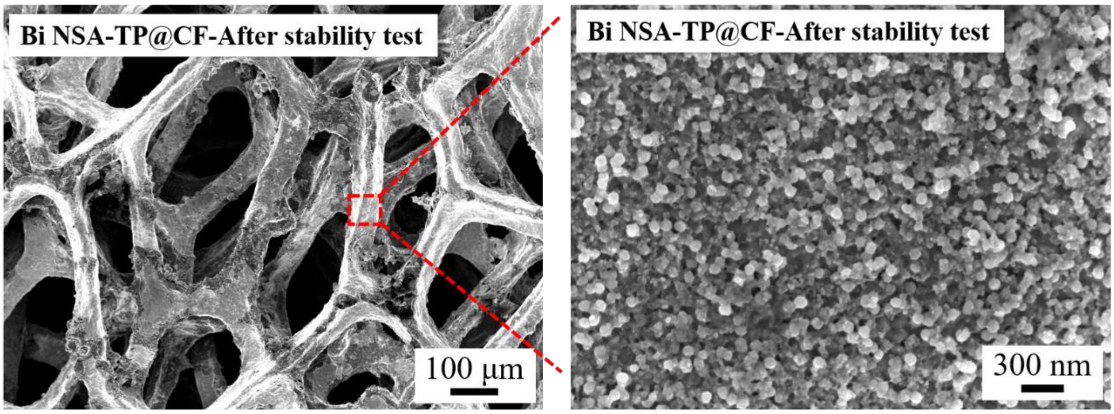

Figure S11 SEM images of Bi NSA-TP@CF after stability test.

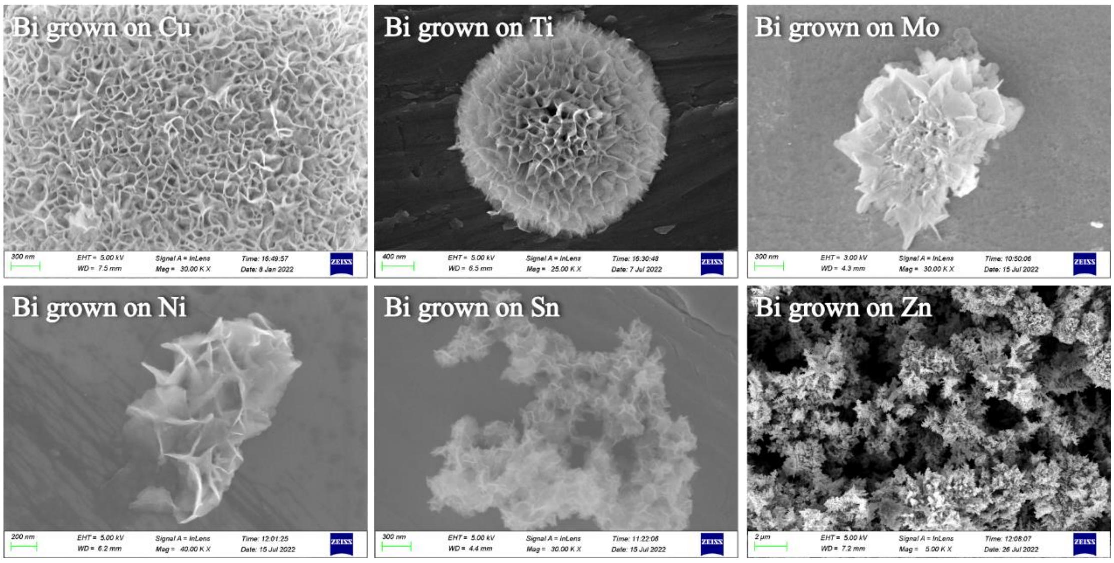

Figure S12 SEM images of Ti foil, Mo foil, Ni foam, Sn foil and Zn foil substrates after same prepare procedure of Bi nanosheets grown on Bi NSA@CF.

To figure out if such galvanic replacement reaction method could be used for Bi nanosheets growth on other substrate materials, several other metal substrates including Ti foil, Mo foil, Ni foam, Sn foil and Zn foil have been investigated. Corresponding standard electrode potentials have been listed below:

$$Bi^{3+} + 3e^{-} \rightleftharpoons Bi \quad 0.31V$$

$$Cu^{2+} + 2e^{-} \rightleftharpoons Cu \quad 0.34V$$

$$Ti^{2+} + 2e^{-} \rightleftharpoons Ti \quad -1.63V$$

$$Mo^{3+}(s) + 3e^{-} \rightleftharpoons Mo \quad -0.2V$$

$$Ni^{2+} + 2e^{-} \rightleftharpoons Ni \quad -0.26V$$

$$Zn^{2+} + 2e^{-} \rightleftharpoons Zn \quad -0.76V$$

More negative potential than $Bi^{3+} + 3e^{-} \rightleftharpoons Bi$, 0.31V indicating the spontaneous property to galvanic replacement reaction for Bi nanosheets growth (Nernstian contribution shift the equilibrium of $3Cu + 2Bi^{3+} \rightleftharpoons 3Cu^{2+} + 2Bi$ and then drive reaction occur). Fig. S12 shows the corresponding SEM images of above-mentioned substrates after the same preparation procedure for Bi nanosheets growth. It can be

seen that although Bi nanostructure can also grow on these substrates, only peculiarly scattered Bi nanosheets or nanodendrites were observed. These phenomena may ascribe to the unmatched ion affinity and reaction energy barrier between the bismuth ions and the substrates during the galvanic replacement reaction. Thus, although the thermodynamic equilibrium potentials are proper, more methodological optimization still needs to be carried out to realize a better growth of nanostructured bismuth catalyst on the above mentioned substrate. Here, we have also tested the CO_2RR performance of Zn substrate based electrode, while it's well known that the Zn catalyst possesses poor activity for CO_2-to-Formate electrochemical reduction[S1, S2]. As shown in Fig. S13, well formate selectivity of about 82.1% was also obtained at the operating potential of -0.9 V vs. RHE, which further revealed the excellent activity of bismuth nanocatalyst for formate formation.

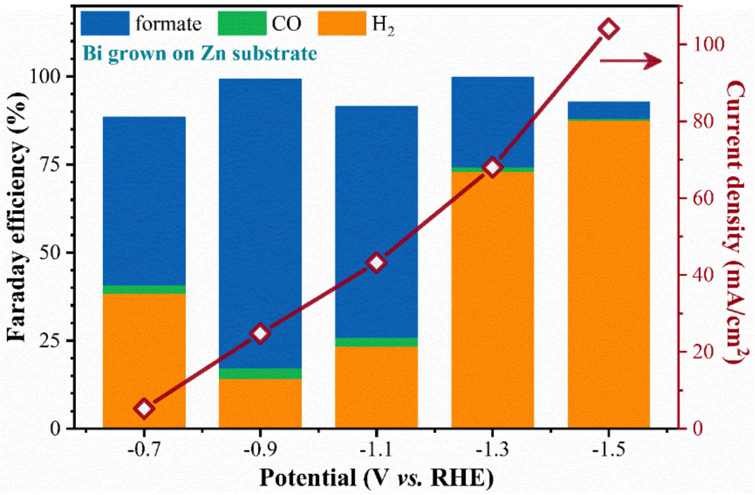

Figure S13 FE distribution and current density of Bi grown on Zn foil substrate at different operating potential

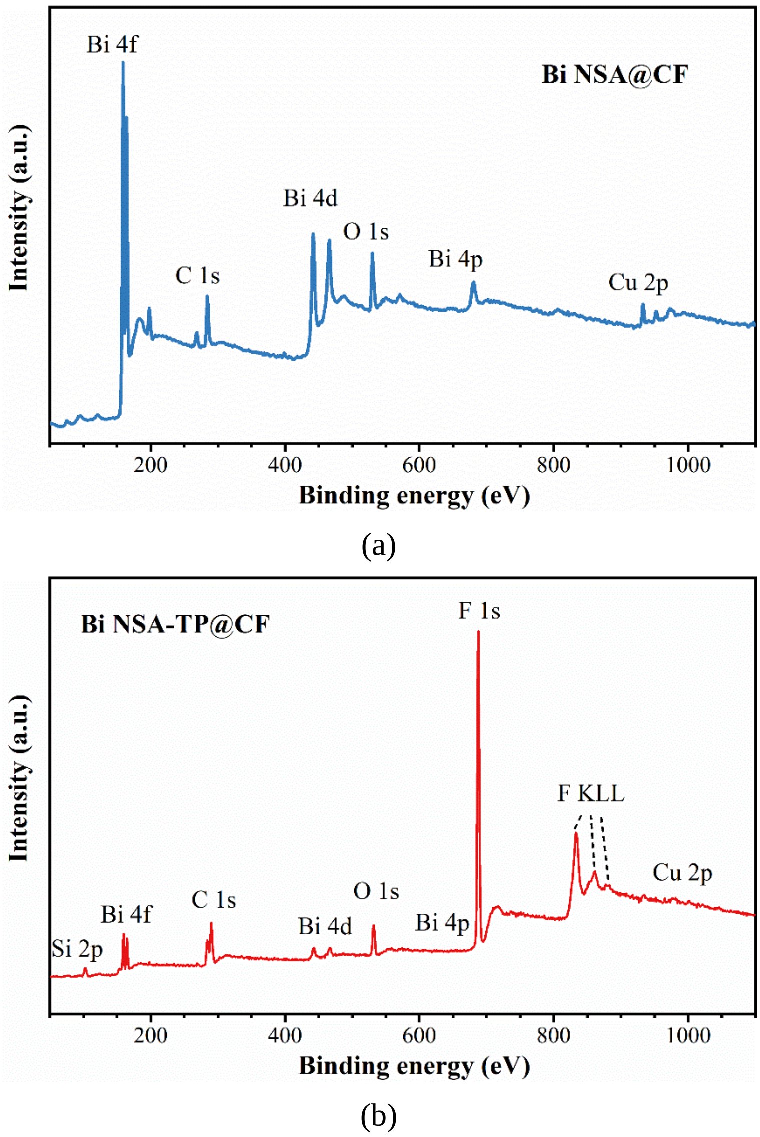

Figure S14 XPS spectra of (a) Bi NSA@CF and (b) Bi NSA-TP@CF electrodes.

SUPPLENMENTARY TABLES

Table S1 CO_2-to-Formate electrochemical performance comparison with previous works in neutral electrolyte.

Catalysts	Electrolyte	Potential (V vs. RHE)	$FE_{formate}$ (%)	Current density (mA/cm^2)	Ref.
Bi NSs	0.5 M $KHCO_3$	-0.58	99	-2.5	[S3]
Bi NSA-TP@CF	0.5 M $KHCO_3$	-0.8	95.31	-14.69	This work
Bi/rGO	0.1 M $KHCO_3$	-0.8	98	-1.85	[S4]
Sn/CN-x	0.1 M $KHCO_3$	-0.9	96	-3.8	[S5]
Bi_2O_3	0.5 M $KHCO_3$	-0.9	91	-8.79	[S6]
Bi NAs/Cu foam	0.5 M $KHCO_3$	-0.95	90	-45	[S7]
Bi NSA-TP@CF	0.5 M $KHCO_3$	-1.0	97.78	-53.34	This work
Wavy SnO_2	0.5 M $KHCO_3$	-1.0	81.4	-25.17	[S8]
Bi nanotubes	0.5 M $KHCO_3$	-1.0	97	-36	[S9]
Bi-Sn Aerogel	0.1 M $KHCO_3$	-1.0	93.9	-9.89	[S10]
SnO_2/Bi_2O_3 oxide	0.1 M $KHCO_3$	-1.0	80	−3.5	[S11]
Bi_5Sn_{60}	0.1 M $KHCO_3$	-1.0	94.8	−35.79	[S12]
Bi NSs	0.1 M $KHCO_3$	-1.1	86	-16.5	[S13]
Sn-Cu alloy	0.5 M KCl	-1.14	82.3	-79	[S14]
$In_{1.5}Cu_{0.5}$	0.1 M $KHCO_3$	-1.2	90	−3.59	[S15]
HB-Cu NW/Sn	0.1 M $KHCO_3$	-1.2	94.17	-17.83	[S16]
S-Bi_2O_3-CNT	0.5 M $KHCO_3$	-1.2	90	-54	[S17]
Bi NSA-TP@CF	0.5 M $KHCO_3$	-1.2	98.15	-94	This work
Bi_2O_3 NSs/MCCM	0.1 M $KHCO_3$	-1.256	93.8	-15.99	[S18]

Table S2 Fitting parameters for the equivalent circuit curves of EIS.

Electrode	R_s (Ω)	R_c (Ω)	R_d (Ω)
Bi NSA@CF	2.87	3.41	4.05
Bi NSA-TP@CF	2.88	2.58	1.95

Table S3 Summary of fitting peaks and atomic ratios of the O 1s spectra for Bi NSA@CF and Bi NSA-TP@CF electrodes.

**Electrode**	**Spectra**	**Component**	**B.E. (eV)**	**Content (at.%)**
Bi NSA@CF	O 1s	Bi-O	530.39	69.54
		Bi-OH	532.69	30.46
Bi NSA-TP@CF	O 1s	Bi-O	530.64	7.57
		Bi-O-Si	531.41	51.88
		Bi-OH	532.62	5.12
		Si-O-Si	533.35	35.44

References

(S1) Jeon, H. S.; Sinev, I.; Scholten, F.; Divins, N. J.; Zegkinoglou, I.; Pielsticker, L.; Cuenya, B. R., Operando Evolution of the Structure and Oxidation State of Size-Controlled Zn Nanoparticles during CO_2 Electroreduction. *J. Am. Chem. Soc.* **2018,** *140* (30), 9383-9386.

(S2) Luo, W.; Zhang, J.; Li, M.; Züttel, A., Boosting CO Production in Electrocatalytic CO_2 Reduction on Highly Porous Zn Catalysts. *ACS Catal.* **2019,** *9* (5), 3783-3791.

(S3) Yang, F.; Elnabawy, A. O.; Schimmenti, R.; Song, P.; Wang, J.; Peng, Z.; Yao, S.; Deng, R.; Song, S.; Lin, Y.; Mavrikakis, M.; Xu, W., Bismuthene for highly efficient carbon dioxide electroreduction reaction. *Nat Commun* **2020,** *11* (1), 1088.

(S4) Duan, Y. X.; Liu, K. H.; Zhang, Q.; Yan, J. M.; Jiang, Q., Efficient CO_2 Reduction to HCOOH with High Selectivity and Energy Efficiency over Bi/rGO Catalyst. *Small Methods* **2020,** *4* (5), 1900846.

(S5) Tian, J.; Wang, M.; Shen, M.; Ma, X.; Hua, Z.; Zhang, L.; Shi, J., Highly Efficient and Selective CO2 Electro-Reduction to HCOOH on Sn Particle-Decorated Polymeric Carbon Nitride. *ChemSusChem* **2020,** *13* (23), 6442-6448.

(S6) Deng, P.; Wang, H.; Qi, R.; Zhu, J.; Chen, S.; Yang, F.; Zhou, L.; Qi, K.; Liu, H.; Xia, B. Y., Bismuth Oxides with Enhanced Bismuth–Oxygen Structure for Efficient Electrochemical Reduction of Carbon Dioxide to Formate. *ACS Catal.* **2019,** *10* (1), 743-750.

(S7) Fan, J.; Zhao, X.; Mao, X.; Xu, J.; Han, N.; Yang, H.; Pan, B.; Li, Y.; Wang, L.; Li, Y., Large-Area Vertically Aligned Bismuthene Nanosheet Arrays from Galvanic Replacement Reaction for Efficient Electrochemical CO_2 Conversion. *Adv. Mater.* **2021,** *33* (35), e2100910.

(S8) Chen, Z.; Fan, T. T.; Zhang, Y. Q.; Xiao, J.; Gao, M. R.; Duan, N. Q.; Zhang, J. W.; Li, J. H.; Liu, Q. X.; Yi, X. D.; Luo, J. L., Wavy SnO_2 catalyzed simultaneous reinforcement of carbon dioxide adsorption and activation towards electrochemical conversion of CO_2 to HCOOH. *Appl. Catal. B-Environ.* **2020,** *261*, 118243.

(S9) Fan, K.; Jia, Y.; Ji, Y.; Kuang, P.; Zhu, B.; Liu, X.; Yu, J., Curved Surface Boosts Electrochemical CO_2 Reduction to Formate via Bismuth Nanotubes in a Wide Potential Window. *ACS Catal.* **2019,** *10* (1), 358-364.

(S10) Wu, Z.; Wu, H.; Cai, W.; Wen, Z.; Jia, B.; Wang, L.; Jin, W.; Ma, T., Engineering Bismuth-Tin Interface in Bimetallic Aerogel with a 3D Porous Structure for Highly Selective Electrocatalytic CO_2 Reduction to HCOOH. *Angew. Chem. Int. Ed.* **2021,** *60* (22), 12554-12559.

(S11) Tian, J.; Wang, R.; Shen, M.; Ma, X.; Yao, H.; Hua, Z.; Zhang, L., Bi-Sn Oxides for Highly Selective CO_2 Electroreduction to Formate in a Wide Potential Window. *ChemSusChem* **2021,** *14* (10), 2247-2254.

(S12) Li, Z.; Feng, Y.; Li, Y.; Chen, X.; Li, N.; He, W.; Liu, J., Fabrication of Bi/Sn bimetallic electrode for high-performance electrochemical reduction of carbon dioxide to formate. *Chem. Eng. J.* **2022,** *428*, 130901.

(S13) Zhang, W.; Hu, Y.; Ma, L.; Zhu, G.; Zhao, P.; Xue, X.; Chen, R.; Yang, S.; Ma, J.; Liu, J.; Jin, Z., Liquid-phase exfoliated ultrathin Bi nanosheets: Uncovering the origins of enhanced electrocatalytic CO_2 reduction on two-dimensional metal nanostructure. *Nano Energy* **2018,** *53,* 808-816.

(S14) Ye, K.; Cao, A.; Shao, J.; Wang, G.; Si, R.; Ta, N.; Xiao, J.; Wang, G., Synergy effects on Sn-Cu alloy catalyst for efficient CO_2 electroreduction to formate with high mass activity. *Sci. Bull.* **2020,** *65* (9), 711-719.

(S15) Wei, B.; Xiong, Y. S.; Zhang, Z. Y.; Hao, J. H.; Li, L. H.; Shi, W. D., Efficient electrocatalytic reduction of CO_2 to HCOOH by bimetallic In-Cu nanoparticles with controlled growth facet. *Appl. Catal. B-Environ.* **2021,** *283*, 119646.

(S16) Yue, P. T.; Fu, Q.; Li, J.; Zhang, L.; Xing, L.; Kang, Z. Y.; Liao, Q.; Zhu, X., Triple-phase electrocatalysis for the enhanced CO_2 reduction to HCOOH on a hydrophobic surface. *Chem. Eng. J.* **2021,** *405*, 126975.

(S17) Liu, S. Q.; Gao, M. R.; Feng, R. F.; Gong, L.; Zeng, H. B.; Luo, J. L., Electronic Delocalization of Bismuth Oxide Induced by Sulfur Doping for Efficient CO_2 Electroreduction to Formate. *ACS Catal.* **2021,** *11* (12), 7604-7612.

(S18) Liu, S.; Lu, X. F.; Xiao, J.; Wang, X.; Lou, X. W. D., Bi_2O_3 Nanosheets Grown on Multi-Channel Carbon Matrix to Catalyze Efficient CO_2 Electroreduction to HCOOH. *Angew. Chem. Int. Ed.* **2019,** *58* (39), 13828-13833.
